# Extracellular vesicles: biomarkers and regulators of vascular function during extracorporeal circulation

**DOI:** 10.18632/oncotarget.26433

**Published:** 2018-12-14

**Authors:** Mark J. McVey, Wolfgang M. Kuebler

**Affiliations:** ^1^ Keenan Research Centre for Biomedical Science, St Michael's Hospital, Toronto, ON, Canada; ^2^ Department of Physiology, University of Toronto, Toronto, ON, Canada; ^3^ Department of Anesthesia, University of Toronto, Toronto, ON, Canada; ^4^ Department of Anesthesia and Pain Medicine, SickKids, Toronto, ON, Canada; ^5^ Department of Surgery, University of Toronto, Toronto, ON, Canada; ^6^ Institute of Physiology, Charité-Universitätsmedizin Berlin, Berlin, Germany; ^7^ German Heart Institute, Berlin, Germany

**Keywords:** extracellular vesicles, extracorporeal circuits, ECMO, cardiopulmonary bypass, hemodialysis

## Abstract

Extracellular vesicles (EVs) are generated at increased rates from parenchymal and circulating blood cells during exposure of the circulation to abnormal flow conditions and foreign materials associated with extracorporeal circuits (ExCors). This review describes types of EVs produced in different ExCors and extracorporeal life support (ECLS) systems including cardiopulmonary bypass circuits, extracorporeal membrane oxygenation (ECMO), extracorporeal carbon dioxide removal (ECCO_2_R), apheresis, dialysis and ventricular assist devices. Roles of EVs not only as biomarkers of adverse events during ExCor/ECLS use, but also as mediators of vascular dysfunction are explored. Manipulation of the number or subtypes of circulating EVs may prove a means of improving vascular function for individuals requiring ExCor/ECLS support. Strategies for therapeutic manipulation of EVs during ExCor/ECLS use are discussed such as accelerating their clearance, preventing their genesis or pharmacologic options to reduce or select which and how many EVs circulate. Strategies to reduce or select for specific types of EVs may prove beneficial in preventing or treating other EV-related diseases such as cancer.

## INTRODUCTION

Use of extracorporeal circuits (ExCor) and extracorporeal life support (ECLS) in medicine has steadily increased over time. Early applications of ExCor/ECLS include hemodialysis (HD), with initial accounts dating back to the nineteen twenties. Cardiopulmonary bypass (CPB) and cardiac and respiratory extracorporeal life support (cECLS and rECLS respectively) systems emerged in the nineteen fifties to seventies. Since then, distinct specialized ECs have been developed to meet growing demands to support critically ill patients, including apheresis machines, ventricular assist devices (VADs), extracorporeal membrane oxygenators (ECMO) and extracorporeal carbon dioxide removal (ECCO_2_R) devices such as the nova-lung and A-lung (Table 1). Examples of critically ill patients benefiting from ExCor and ECMO include both solid organ and hematological oncology patients [1, 2]. Though typically utilized in hospitals, a growing trend towards home HD programs or enhanced portability of ExCor/ECLS such as VADs has allowed patients to leave hospital and increased patient mobility opening the possibility of long term destination therapy for conditions such as heart failure with use of prolonged-ECLS in the form of VADs [3, 4]. As such, the use of an increasingly complex range of ExCor/ECLS is expanding in terms of the number of patients utilizing this evolving resource both in and out of the hospital.

**Table 1:** Extracorporeal circuit types describing specific indications, circuit functions, and components

Circuit	Indication	Function	Components
**HD**	renal failure acidosis electrolyte abnormality toxins fluid overload uremia	removal of toxins modification of circulating volume	cannulas/needles dialyser pressure monitors anticoagulant pump ultra-filtration unit filters heaters/heat exchangers de-aerator pH monitors
**CPB**	cardiac/pulmonary surgery complex vascular surgery airway surgery brain surgery deep hypothermic circulatory arrest rewarming	selective exclusion of cardiac and vascular structures from circulation oxygenation and CO_2_ removal	cannulas oxygenator heater CO_2_ exchanger suction/vents pump (roller/centrifugal) blood storage chambers hemo-concentrator cardioplegia system bubble detector/trap anesthetic delivery unit/gas blender blood reservoirs
**apheresis**	immune therapy blood donation neurologic diseases hematologic diseases metabolic diseases dermatologic diseases rheumatologic diseases renal diseases intoxications neoplastic diseases (photopheresis)	isolation and sequestration of blood components photopheresis therapy	cannulas blood pump plasma pump plasma separator lipo-sorber membrane filters centrifugal components blood warmer anticoagulants regeneration solutions/pumps valves pressure sensors
**VAD**	heart failure bridge to transplant destination therapy	partial or total cardiac support	cannulas pump (impellar) Filters valves
**ECCO_2_R**	pulmonary hypertension hypercapnia high airway pressures respiratory failure/fatigue weaning from mechanical ventilation bridge to transplant	oxygenation and CO_2_ removal independent of the lungs and pulmonary circulation	cannulas oxygenator CO_2_ exchanger (anticoagulant-coated diffusion membrane)
**ECMO**	Refractory cardiopulmonary dysfunction bridge to transplant arrhytrmia sepsis drug overdose PE anaphylaxis failure to wean CPB	partial or total cardiac support and oxygenation and CO_2_ removal	cannulas oxygenator/membrane heat exchanger membrane filtration pump CO_2_ exchanger reservoir anticoagulant pressure monitors

CO_2_: carbon dioxide; CPB: cardiopulmonary bypass; ECMO: extracorporeal membrane oxygenation; HD: hemodialysis; PE: pulmonary embolism; VAD: ventricular assist device.

As ExCor/ECLS use rises within increasingly diverse patient populations (including oncology patients) there is a growing need to develop biomarkers capable of predicting ExCor/ECLS associated adverse events. Available biomarkers are currently limited at predicting ExCor/ECLS adverse events. Notably, ExCor/ECLS leads to generation of elevated blood levels of extracellular vesicles (EVs). EVs are emerging as potential biomarkers of disease. This review aims to characterize EVs associated with use of different ExCor/ECLS systems and discuss their potential roles as biomarkers and effectors of ExCor/ECLS associated adverse events. Lastly, the use of ExCor as a strategy for therapeutic manipulation of EVs will be discussed such as systems capable of reducing or selecting specific EVs.

## REVIEW METHODOLOGY

Studies included in this narrative review examining available literature on EVs and extracorporeal support (ExCor/ECLS) were obtained by searching the PubMed electronic database during 2016–2018 using the following keywords: extracorporeal, ECMO, ECLS, hemodialysis, Novalung, cardiopulmonary bypass, cell saver, VAD, LVAD, BIVAD, RVAD, ventricle assist device, artificial heart, extracellular vesicles, microparticles, microvesicles, apoptotic bodies, exosomes. Searched review- and original-papers were excluded if they did not meet the following inclusion criteria: being published in English (or if translation to English was available) as well as having relevant titles and abstracts. The goal of this review is to introduce physicians (e.g. intensivists, oncologists) and scientists to the relevance and potential of EVs to influence patient care during ExCor/ECLS.

## INDICATIONS FOR EXCOR/ECLS USE

Use of ExCor/ECLS has been revolutionary in supporting a wide range of previously fatal conditions such as end stage cardiopulmonary diseases and renal failure (Table 1) [5, 6]. ExCor/ECLS use can be short-term for minutes to hours such as for cardiopulmonary bypass or longer such as days as seen with hemodialysis or VADs and finally long-term or destination use such as with prolonged-ECLS using VADs [7, 8]. There is an ever-increasing number of different extracorporeal circuits entering clinical use (examples shown in Table 1) which corelates with increasing use of ExCor/ECLS with patients [9–13]. Though the interaction of ExCor/ECLS with the patients’ physiological functions is intended to be supportive, due to their intimate connection with the patient, ExCor/ECLS can unfortunately lead to serious adverse events.

## EXCOR/ECLS ASSOCIATED ADVERSE EVENTS

Compared with the human circulation (without ExCor/ECLS use), interaction with distinct ExCor/ECLS systems create altered physiologic conditions for circulating cells including foreign surfaces, increased shear forces, perturbations in the flow of blood (pulsatile or continuous) and altered function of circulatory cells due to circuit components and use of medications related to ExCor/ECLS use such as anticoagulants (Tables 2–3). ExCor/ECLS adverse events are influenced in part by patient related factors such as pre-existing co-morbid disease states, medications being consumed, inflammation and circuit related factors such as the components within ExCor/ECLS systems (Tables 2–3). Specifically, within circuits, there are components which add risk for adverse events such as cannulas and tubing whose foreign material activates and reduces levels of circulating adhesive cells such as platelets or membrane oxygenators which through non-physiological shear forces traumatise RBCs leading to hemolysis. Specific components within ExCor/ECLS circuits such as oxygenators create local regions with high shear stresses (8.4 N/m^2^) [14]. The consequences of blood flowing through such high shear environments depend in part on the magnitude of the shear rate, exposure time and the cells involved. Platelets exposed to high shear stresses for as little as 7 milliseconds (ms) release procoagulant phospholipids and after a 113 ms exposure platelets no longer respond to ADP agonist stimulation [15]. These physiologic changes can culminate in adverse events directly within the circulation and proximal organs (Table 2). With so many potential harms for patients it is important to monitor the proper function of ExCor/ECLS systems and detect, predict, and – ideally – prevent or reduce adverse effects of ExCor/ECLS use which may lead to morbidity or even mortality.

**Table 2:** Extracorporeal circuit related adverse events and associated mechanisms of action

Adverse event	Mechanism of action	References
anemia	contact with foreign material (circuit tubing/cannulas) increased shear forces decreased red blood cell deformability hemolysis	[105]
thrombocytopenia	platelet activation platelet sequestration	[15, 106]
coagulopathy	loss/dysfunction of von Willebrand Factor, fibrinogen, complement factors, coagulation factors requirement for anticoagulation (e.g. heparin) disseminated intravascular coagulopathy	[21, 25, 61, 107]
inflammation	increased endothelial permeability increased susceptibility to infection reactive oxygen species myeloperoxidase enzyme activity complement activation thrombin elastase neutrophil extracellular	[21, 108]
cardiac dysfunction	inflammatory cytokine release arrythmia myocardial infarction	[56, 108]
pulmonary injury	cell free hemoglobin	[109]
gastrointestinal bleeding	abnormal cell growth – arterio-venous malformations continuous as opposed to pulsatile flows	[3, 25, 110]
brain injury	cannula positioning leading to ischemia embolic/hemorrhagic stroke	[3, 21, 25]
kidney injury	low flow states cardio-renal syndrome	[21, 56]

**Table 3:** Extracorporeal circuits (ECLS/ExCor systems) lead to changes in systemic flows, shear stresses and may require anticoagulation

Circuit	Systemic flows	Shear stresses possible with ExCor/ECLS use	Systemic anticoagulant
**normal circulation** **(no ExCor/ECLS)**	pulsatile, 2.6–4.2 L/min/m^2^	straight arteries laminar flow 1.5-1.68 N/m^2^ [111, 112]	no
**HD**	pulsatile 0.5-0.8 L/min [113]	114 N/m^2^ [114]	yes
**CPB**	continuous or pulsatile 1.75 to 3.5 L/min/m^2^	994 N/m^2^ [115]	yes
**apheresis**	pulsatile 0.06-0.24 L/min [116]	not reported	yes
**VAD**	continuous or pulsatile 3.5–10 L/min [117]	80-300 N/m^2^ [22, 118]	yes
**ECCO^2^R**	pulsatile 1-15 L/min [119]	not reported	no
**ECMO**	continuous 4-8 L/min	2 N/m^2^ [120]	yes

CPB: cardiopulmonary bypass; ECMO: extracorporeal membrane oxygenation; HD: hemodialysis; VAD: ventricular assist device.

## AVAILABLE BIOMARKERS FOR EXCOR/ECLS RELATED ADVERSE EVENTS

Having an accurate means of monitoring detrimental effects is of crucial importance to maximize benefits while preventing adverse events related to ExCor/ECLS use. Though potentially useful, the available and emerging ExCor/ECLS biomarkers (Table 4) provide an incomplete picture of endothelial function, coagulopathy or degree of inflammation present and therefore highlight the need for further biomarker research.

**Table 4:** Biomarkers predicting adverse events during extracorporeal circuits

Adverse event	Biomarker
**hemolysis**	↓ haptoglobin [121] ↑ free-hemoglobin [3, 122] ↑ fibrin stranding ↓ hemoglobin
**inflammation**	↑ platelet – leukocyte aggregates, NETS [56, 108]
**ischemia reperfusion**	↑ lipid peroxidation [123] ↑ protein carbonylation [123]
**endothelial dysfunction**	↑ circulating endothelial cells [59, 71, 124] ↑ circulating endothelial cell progenitors [58, 71, 124] ↑ circulating soluble E-selectin [25]
**platelet activation**	↑ sCD40L [25] ↑ circulating soluble P-selectin [25, 56] ↓ platelet count ↓ platelet function testing
**coagulation**	↑ TF [25] ↓ large vWF multimers [3, 25] ↓AT3,
**infection**	↑ procalcitonin [125] ↑ CRP
**mortality**	↑ ALT/billirubin [126] ↓ urine output [127]

ALT: alanine aminotransferase; AT3: anti-thrombin III; CPB: cardiopulmonary bypass; CRP: C reactive protein; TF: tissue factor; vWF: von Willebrand factor.

## EXTRACELLULAR VESICLES

EVs are rapidly emerging candidates to address the gap of reliable biomarkers for monitoring ExCor/ECLS adverse events. EVs are a global designation for a range of small (<1 μm) cell derived lipid vesicular structures with differing sizes, compositions and origins. Within the EV hierarchy, vesicles can be discriminated in part based on specific triggers for their formation and the composition of their membranes and intravesicular cargos (reviewed in part in [16, 17]). EVs can be further sub-divided by size as apoptotic bodies that are larger than microparticles (MPs) /microvesicles (MVs) which are in turn larger than exosomes respectively. First, apoptotic bodies are formed during apoptosis and are physically large EVs typically < 400 nm–1000 nm rivalling platelets in terms of size, but unlike platelets contain histones and genomic DNA. Apoptotic bodies are formed from cellular fragmentation during apoptosis. Second, MP/MV sized EVs typically form from sections of externalized inner cell lipid bilayer membrane involving lipid rafts, especially under conditions of cellular stress or activation and are typically 100-1000 nm). Under usual conditions, membrane asymmetry is maintained by enzymes such as flippases, floppases and scrambles, yet when EVs are formed the activity of these enzymes is altered, in part due to increased intracellular calcium which culminate in cellular contraction, membrane lipid changes and blebbing of EVs [17]. EVs can originate from many different types of parent cells such as: endothelial cells (e-EVs), endothelial progenitor cells (EPC-EVs), leukocytes (l-EVs), platelets (p-EVs), erythrocytes (r-EVs), smooth muscle cells (smc-EVs), monocytes (m-EVs) and granulocytes (neutrophils; g-EVs). MPs can be studded with outer membrane surface markers which in part facilitate characterizing their parent cells of origin. Third, exosomes form from extracellular release of intracellular mutivesicular bodies and are typically smaller, commonly less than 100 nm in diameter. As opposed to MPs, exosomes express a more concentrated endowment of heat shock proteins, tetraspanins such as CD63 or CD9 and have considerable acetyl-cholinesterase activity [18]. Exosomes are thought to form intracellularly at multivesicular bodies which merge and are released outside the cell membrane [17]. EVs such as MPs are of interest in an ever-increasing number of research fields (such as oncology) due to their potential duality of being biomarkers but also potential effectors of disease [19]. EVs are of interest as they may mediate physiological changes occurring with ExCor/ECLS use, as will be described later this review. Emerging strategies of modify EV-functions including: reducing EV numbers or selectively remove detrimental- or increasing beneficial-EVs as well as pharmacological strategies to modify EVs to make them less injurious will be discussed in the review. This review will predominantly discuss the interactions of EVs primarily resembling MP/MVs in relation to ExCor/ECLS as the literature is essentially devoid of descriptions of ExCor/ECLS effects on apoptotic bodies or exosomes.

## EVS ARE ASSOCIATED WITH EXCOR/ECLS USE

EVs are plausible candidate biomarkers for ExCor/ECLS adverse events due to their association with ExCor/ECLS use. As we discuss later in detail, a challenge affecting the interpretation of many EV-ExCor/ECLS studies is the general problem of differentiating between EV formation due to ExCor/ECLS as opposed to disease progression. A range of ExCor/ECLS systems including CPB, ECMO, VADs, HD and plasmapheresis systems have been shown to be associated with elevated levels of EVs. In addition to ExCor/ECLS systems, their adjuncts, such as pericardial suction may be rich sources of EVs - up to ten-fold higher than pre-operative blood levels [20, 21] - which in certain cases get re-transfused to the patient. The number and types of EVs depends at least in part on the different components found in various circuits (Table 1). The scarcity of EV studies related to individual components within ECLS/ExCor systems necessitates that for the purpose of the present review, we have primarily presented EV studies associated with ECLS/ExCor modalities rather than circuit components.

**Figure 1:**
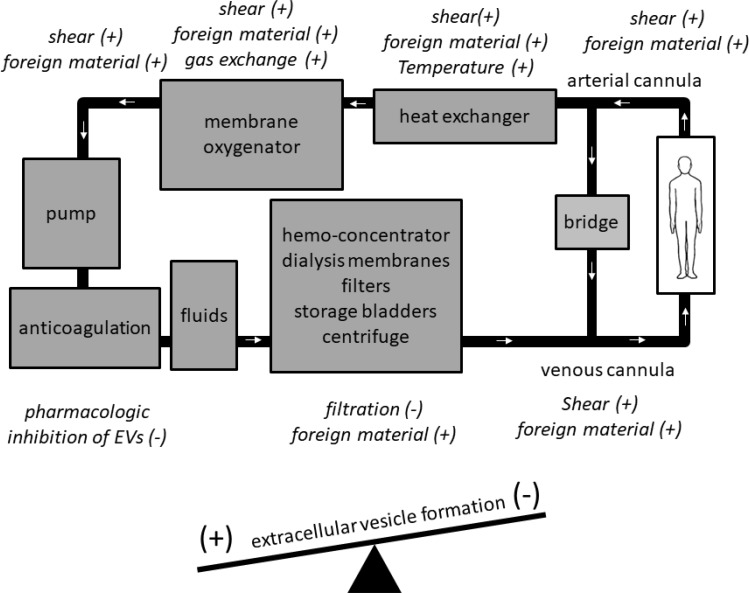
Specific components of extracorporeal circuits have the potential to increase (+) or decrease (−) extracellular vesicle (EV) levels

Within individual ExCor/ECLS systems, EV numbers and subtypes may vary depending on the specific design aspects of certain ExCor/ECLS systems leading to increased shear stresses and thus, to EV formation (Figure 1). For example, high shear (up to 300 N/m^2^) models simulating ExCor/ECLS cause increased formation of p-EVs [22]. When present in ExCor/ECLS systems, oxygenators are a specific site of EV generation as p-EV levels were shown to be higher post- as compared to pre- oxygenator [23]. Beyond the oxygenator, pumps which actively propel blood through ECLS systems can lead to variable EV formation. Using *in vitro* swine blood, a recent study demonstrated higher p-EVs in centrifugal compared with roller-head pump systems at comparable flows of 0.3 L/minute [24]. Importantly, susceptibility of EV formation seems to depend on the cell types exposed to ExCor/ECLS systems. Within elevated shear stress environments such as seen with ExCor/ECLS use, platelets are considerably more vulnerable to activation and fragmentation at elevated shears (10–25 N/m^2^) than erythrocytes or leukocytes [25–27]. The shear-induced generation of p-EVs in ExCor/ECLS is considered to occur analogous to altered circulatory flow conditions in other non- ExCor/ECLS contexts, as e.g. mitral valve disease has been shown to have twice the circulating p-EV levels compared to normal controls [28]. Beyond platelets, other cell types such as the endothelium have shown associations with EV formation under the altered flow conditions imposed by ExCor/ECLS. Part of the endothelial response to altered flow within the circulation is to increase endothelial-EV populations. An example of this concept is an experiment with a blood pressure cuff inflated to 220 mmHg for 20 minutes on one arm showed acute increases in e-EVs relative to the other arm with no cuff in healthy volunteers [29]. Though in this instance transient ischemia then reperfusion may also have additionally contributed to increased e-EVs which is different from typical extracorporeal circuit flows. Beyond altered shear rates, colder temperatures are associated with higher EV levels [27, 30] which are pertinent for ExCor/ECLS systems such as CPB during bypass or deep hypothermic circulatory arrest. As such ExCor/ECLS use often leads to higher levels of EVs of different parental cell origins, to differing degrees depending on the individual components of the ExCor/ECLS systems in question.

## EVS INCREASE DURING THE USE OF EXCOR/ECLS

Studies examining EVs in ExCor/ECLS have shown differences in EV numbers and subtypes over time (Tables 5–8). The half-life of EVs in the circulation is not well defined under physiological conditions let alone in conjunction with ExCor/ECLS use. Published estimates describe short circulating half-lives of five to ten minutes for p-EVs in the circulation of rabbits [31, 32]. Observations of p-EVs kinetics in humans receiving exogenous EVs from blood products show EVs may circulate longer in the order of hours [33]. This is of relevance considering many patients with ExCor/ECLS require transfusion, which may be a confounding source of EVs when EVs are used as a biomarker. ExCor/ECLS may further complicate kinetic measurements of EVs as they may adhere to the foreign material of the ExCor/ECLS systems. Despite possible sequestration of EVs on foreign materials, or increases related to transfusion, there is a distinct increase in EVs associated with initiation of ExCor/ECLS use. In a pig CPB model, increased p-EVs were evident as early as 10 minutes after onset of CPB [23]. Just as there is a rapid onset, there also appears to be a fairly rapid temporal resolution of elevated p-EV levels post CPB in less than 1 hour [23, 34]. The transient nature of the EV response is in agreement with dynamic changes in EVs seen with other vascular stressors such as percutaneous coronary catheter procedures which trigger transiently increased levels of circulating e-, p- and r-EVs for around an hour due to endothelial traumatization by vascular sheaths and wires (with instrumentation and tubing notably resembling what is required for insertion and maintenance of ExCor/ECLS) but return to lower pre-procedure levels after one hour [35]. Notably, however, increases in p-EVs during CPB have not been unanimously confirmed by all studies, as e.g. a study examining 71 patients requiring CPB showed signs of complement activation but no accumulation of p-EVs post CPB [36]. The lack of increase in this specific study may, however, be attributable to the sole measurement of phosphatidylserine (PS+) EVs which would not have captured non-PS+ EV increases over time from CPB.

**Table 5:** Cardiopulmonary support with extracorporeal circuits (ECLS/ExCor systems) includes CPB and ECMO

Circuit type *Observations*	*N*	CARDIAC EV phenotype	EV isolation technique	Detection technique	Population	Reference
**CPB** ***pericardial blood contains elevated levels of EVs***	13	C1q C4 C3 complement bound EVs	citrated plasma 1550 g × 20 minutes −80 C thawed 19000 g × 30 minutes	FCM	adult cardiac surgery	[21]
**CPB** ***iNO and PGI2 reduced p-EV levels***	41	CD42 p-EVs	citrated plasma	FCM	adult cardiac surgery	[61]
**CPB** ***transient CPB increased p-EVs***	12	CD61 CD62 p-EVs	citrated plasma	FCM	pigs	[23]
**CPB** ***transient CPB increased p-EVs***	18	CD61 CD62 p-EVs	citrated plasma	FCM	pigs	[128]
**CPB** ***lower levels of EVs in CPB compared with suctioned pericardial blood***	2 females 8 males	Annexin V EVs	citrated plasma 3000 g × 10 minutes 13000 g × 2 minutes	ELISA	adult cardiac surgery	[20]
**Neonatal (ECMO)** ***Jostra-rotaflow-HL20*** ***Centrifugal ECMO created more p-EVs than roller pump ECMO***	12	CD61 p-EVs	heparinized blood 300 g × 10 minutes post staining	FCM	*in vitro* pig	[24]
**CPB** ***increased EV levels in pericardial blood compared with systemic blood***	6	Annexin V EVs CD61 CD42 p-EVs CD235 r-EVs CD14 m-EVs	citrated plasma 1550 g × 15 minutes	FCM *In vitro* Thrombin assays	adult cardiac surgery	[72]
**CPB** ***cell saver removed EVs***	13	Annexin V EVs CD235 r-EVs CD61 p-EVs	citrated plasma 1550 g × 20 minutes 17570 g × 30 minutes	FCM	adult cardiac surgery	[75]
**CPB** ***pericardial blood has elevated EVs which upon retransfusion do not persistently stay elevated in circulation***	2 females 11 males	Annexin V EVs CD235 r-EVs CD61 p-EVs	citrated plasma 1550 g × 20 minutes 17570 g x 30 minutes	FCM	adult cardiac surgery	[129]
**CPB** ***complement complexes were elevated post CPB while p-EVs were not***	3 females 68 males	Annexin V EVs CD61 p-EVs	citrated plasma 1550 g × 20 minutes 17570 g × 30 minutes	FCM	adult cardiac surgery	[36]
**ECMO** ***in vitro experiments involving prevention of platelet activation show decreased p-EV levels in ECMO circuits after 2 hours***	10 patients	CD42 p-EVs	citrated plasma 100 g × 10 minutes 1500 g × 10 minutes	FCM	*in vitro* human	[88]

CPB: cardiopulmonary bypass; ECMO: extracorporeal membrane oxygenation; EV: extracellular vesicles; FCM: flow cytometry; m-EV: monocyte-EV; p-EV: platelet-EV; r-EV: red blood cell associated EV.

**Table 6:** Cardiopulmonary support with extracorporeal circuits (ECLS/ExCor systems) can involve VADs

Circuit type *Observations*	*N*	VAD EV phenotype	EV isolation technique	Detection technique	Population	Reference
**LVAD** **HMII, HVAD** ***no change in p-EVs***	3 females 17 males	CD62p CD41 CD42 p-EVs	PFA fixed PPP	Not disclosed	adult LVAD patients	[44]
**LVAD** ***Ventrassist IRBP led to more l-EVs than Rotaflow CP***	15 samples	CD45 l-EVs	CPDA cow blood	EM FCM Imaging FCM	*in vitro*	[37]
**LVAD** **Centrimag CP** ***increases l-EVs***	15 samples	CD45 l-EVs	CDPA cow blood	FCM	*in vitro*	[3]
**LVAD** **HMII, Thoratec VAD** **Ventrassist, Circulite, ECMO** ***increases p- and l-EVs***	3 females 9 males	CD31 CD61 p-EVs CD11b l-EVs CD62e e-EVs	citrated plasma 1550 g × 15 min	FCM	adult LVAD patients	[42]
**LVAD** **HMII** ***lower EVs after 3 months post-VAD***	5 females 25 males	Annexin V EVs	not disclosed	ELISA Zymutest EV-activity test	adult LVAD patients	[40]
**LVAD** **HMII** ***EVs higher in patients with adverse events***	17 patients	Annexin V EVs	not disclosed	FCM	adult LVAD patients	[56]
**LVAD** **HMII** ***no differences in EV levels before and 3 months after LVAD insertion***	8 males	Annexin V EVs	not disclosed	ELISA Zymutest EV-activity test	adult LVAD patients	[39]
**LVAD** **HeartWare continuous flow** ***elevated p-EVs after 1 year*** ***4 patients had BiVAD***	3 females 25 males	lactadherin CD41 p-EVs	citrated PPP	FCM	adult LVAD patients	[43]
**LVAD** **HMII continuous flow** ***though not statistically significant lower EVs 3 months post-VAD which returned to baseline at 6 months post-VAD***	5 females 18 males	Annexin V EVs	not disclosed	ELISA	adult LVAD patients	[41]
**LVAD** **HeartWare** ***increased EVs with LVAD***	10 patients	CD41 Cd31 p-EVs CD45 l-EVs CD235 r-EVs CD62 CD144 e-EVs CD31 e-EVS	citrated plasma 300 g × 15 min, 10000 g × 5 min twice, 30000 g × 90 min 4 ° C	FCM ELISA	adult LVAD patients	[38]

CPDA: citrate phosphate dextrose adenine; e-EV: endothelial-EV; EM: electron microscopy; EV: extracellular vesicles; FCM: flow cytometry; HM: HeartMate; l-EV: leukocyte-EV; PFA: paraformaldehyde; p-EV: platelet-EV; PPP: platelet poor plasma; r-EV: red blood cell derived EV.

**Table 7:** Renal support with extracorporeal circuits (ExCors) includes HD

Circuit type *Observations*	*N*	Dialysis EV phenotype	EV isolation	Detection technique	Patient population	Reference
**CVVH** ***in vitro use of CVVH can remove e-EVs***	n/a	Annexin V EVs CD31 e-EVs	cell culture 145 g × 8 minutes 100000 g × 6 minutes	FCM	*in vitro* Endothelial cells	[74]
**HD** ***dialysis dependent patients have higher levels of e-, r- and p-EVs. e-EV levels correlated with vascular function tests***	19 females 25 males	Annexin V EVs CD31 CD144 e-EVs CD235 r-EVs CD41 p-EVs CD3 CD45 l-EVs Cd11b m-EVs CD66b g-EVs	citrated plasma 11000 g × 2 minutes 13000 g × 45 minutes 20500 g x 20 minutes	FCM	adult ESRD patients	[67]
**HD** ***EMP levels correlated with cardiovascular risk in ESRD patients***	30 females 51 males	Annexin V CD31 CD144 e-EVs CD11b l-EVs CD41 CD31 p-EVs CD235 RMP	citrated plasma 500 g × 15 minutes 14000 g × 5 minutes	FCM	adult ESRD patients	[55]
**HD** ***shear stress varies inversely to EMP levels***	14 females 20 males	CD41 CD31 p-EVs CD31 CD144 e-EVs	citrated plasma 11000 g × 2 minutes 13000 g × 45 minutes 20500 g × 20 minutes	FCM	adult ESRD patients	[78]
**HD** ***g- and p-EVs increased with HD***	22 females 18 males	CD41 p-EVs CD66b g-EVs	citrated plasma 150 g × 20 minutes 300 g × 20 minutes 15000 g × 30 minutes with EDTA treatment	FCM	adult ESRD patients	[48]
**HD** ***e-EV levels were higher in HD patients compared with pre-HD or PD patients***	6 females 6 males	CD144 CD146 e-EVs	citrated plasma 1500 g × 15 minutes 13000 g × 2 minutes	FCM	pediatric ESRD patients	[52]
**HD** ***patients having HD had elevated levels of all EVs examined. Patients had elevations in p-EVs after HD sessions***	14 females 16 males	Annexin V EVs CD41 p-EVs CD144 CD146 e-EVs CD45 l-EVs	citrated plasma 1500 g × 15 minutes 13000 g × 2 minutes	FCM	adult ESRD patients	[46]
**HD** ***patients having HD show indirect evidence of increased p-EVs levels during dialysis***	4 females 3 males	CD41 CD62 p-EVs	citrated plasma 180 g × 20 minutes 13000 g × 2 minutes	FCM	adult ESRD patients	[100]
**HD** ***patients having HD had elevated levels of e-EVs which correlated with endothelial progenitor cell levels***	18 females 20 males	CD31 e-EVs	citrated plasma 1500 g × 15 minutes 13000 g × 2 minutes	FCM	adult ESRD patients	[47]
**HD** ***patients having HD have elevated e-EV levels in comparison to non-HD ESRD and control patients***	11 females 4 males	Annexin V EVs CD31 e-EVs	citrated plasma 13000 g × 5 minutes	FCM	adult ESRD patients	[51]
**HD** ***on-line hemofiltration for HD patients may reduce e-EV levels***	4 females 11 males	Annexin V EVs CD31 e-EVs	citrated plasma 13000 g × 5 minutes	FCM	adult ESRD patients	[60]
**HD** ***patients having HD have elevated levels of* EVs**	5 females 5 males	Annexin V EVs CD41 CD62p CD63 CD61 p-EVs CD235 r-EVs CD144 CD62e CD54 CD106 e-EVs CD45 CD66e CD20 CD8 CD4 CD15 l-EVs CD66b g-EVs CD14 m-EVs TF EVs	citrated plasma 1550 g × 20 minutes	FCM EV thrombin generation assay	adult ESRD patients	[49]
**HD** ***patients having HD or PD have elevated levels of pro-coagulant EVs***	10 females 10 males	CD144 e-EVs CD42b p-EVs	citrated Plasma 1500 g × 15 minutes twice then 13000 g × 2 minutes then 18000 g × 30 minutes	NTA WB EM EV thrombin generation assay	adult ESRD patients	[50]
**HD** ***reductions in* e-EVs *were greater with HFR than online hemofiltration***	7 females 10 males	CD31 e-EVs Annexin V EVs	not disclosed	FCM	adult ESRD patients	[53]
**HD** ***increased* p- and e-EVs *vascular access failure which correlates with ExCor stenosis***	52 females 30 males	CD31/CD51 e-EVs CD31 CD41 p-EVs	citrated plasma 160 g × 5 min 4° C then 1200 g × 6 min 4° C	FCM	adult ESRD patients	[45]

CVVH: continuous veno-veno hemofiltration; EM: electron microscopy; e-EV: endothelial-EV; EV: extracellular vesicle; FCM: flow cytometry; g-EV: granulocyte-EV; HD: hemodialysis; HFR: hemofiltration with endogenous reinfusion; l-EV: leukocyte-EV; m-EV: monocyte-EV; NTA: nanoparticle tracking analysis; PD: peritoneal dialysis; p-EV: platelet-EV; r-EV: red blood cell derived EV; WB Western Blot.

**Table 8:** Apheresis circuits are extracorporeal circuits (ExCors) indicated for a wide range of diseases including intoxications and autoimmune conditions

Circuit type *Observations*	*N*	EV phenotype	Apheresis EV isolation	Detection techniques	Patient population	Reference
**apheresis *circuit*** ***lowered EVs***	9 males 3 females	Annexin V EVs CD41 p-EVs CD144 e-EVs CD11b m-EVs CD235 r-EVs	citrated plasma 1509 g × 10 min 100 000 g × 60 min	NTA TRPS FCM EV fatty acids EV IIa generation	adult FH patients	[76]
**leukopheresis** ***increased* p-EVs**	8 males 4 females	CD42a p-EVs	citrated plasma 200 g × 10 min 1000 g × 15 min	FCM	adult malignant lymphoma patients	[130]
**leukopheresis** ***decreased* p-EVs *and increased g-EVs***	6 females	Annexin V EVs CD61 CD42a p-EVs CD66 CD16 g-EVs	EDTA plasma 1600 g × 20 min −70 C freeze, thawed 17000 g × 20 min	EM FCM	adult RA patients	[80]
**plasmapheresis** ***increased* p-EVs**	6 males 6 females	PAC-1 CD62p CD61 p-EVs	citrated plasma	FCM	adult volunteers	[131]
**plateletpheresis** ***produce differing levels of* p-EVs *in their platelet concentrates***	42 platelet samples	CD61 p-EVs	citrated plasma	FCM NTA	adult donors	[83]

EM: electron microscopy; Extracellular vesicle (EV); FCM: flow cytometry; FH: familial hypercholesterolemia; g-EV: granulocyte-EV; m-EV: monocyte-EV; NTA: nanoparticle tracking analysis, p-EV: platelet-EV; RA: rheumatoid arthritis; r-EV: red blood cell derived EV; TRPS: tunable resistive pulse sensing.

Trends of increasing EV numbers over time were seen at 6 and 24 hours in *in vitro* tests involving prolonged-ECLS such as VADs (VentrAssist IRBP, RotaFlow CP and CentriMag) under constant hemodynamic conditions and flows (5 L/min) [3, 37] (Table 6). While there is general consensus that VADs cause an acute increase in circulating EV levels, long-term effects are more variable. At 3 months post-VAD insertion, r-, l- and e-EVs were found to be increased in patients with HeartWare LVADs compared to VAD free controls [38]. Data on EV numbers at 3 months post VAD insertion are, however, not consistent in the literature in as much as other studies found no difference in PS+ EVs after 3 months in comparison to pre-VAD baseline levels [39] while a study of 30 patients with HEART MATE II units showed decreased PS+ EV levels at 3 months in comparison to pre-VAD placement [40]. The authors of these studies showing unchanged or lower EV numbers theorized patients likely have improved hemodynamics post-VAD implantation. Another possible explanation for these discrepancies again lies in methodological differences of the latter two studies which only examined PS+ EVs, which represent only a fraction of the total EVs found in the circulation. An additional study examining LVADs showed a reduction in EV numbers at 3 months post-insertion compared with baseline at time of VAD placement which then returned to baseline levels at 6 months post insertion [41]. Though the changes were not statistically significant, in this study the authors theorized that initially at 3 months EVs may have fallen due to improved perfusion, yet after 6 months the potentially high shear rates may have accelerated EV production back to levels similar to baseline. Interpretation of this study in comparison to others is challenging due to the absence of a control group (non-VAD patients) and lack of phenotypic investigation of the EV populations beyond enumeration of PS expressing EVs. Yet a study of 12 patients on extracorporeal assist devices for approximately 5 months, of which 11 had VADs showed elevated counts of p-, l- and e-EVs compared with control patient who did not have these devices which corroborates a longer-term increase in EVs (<3 months) [42]. Finally, extrapolating the general trend of increasing number of EVs over time seen in studies ranging from seconds, hours to months, after a year or longer two studies have found elevations in p-EVs relative to baseline initiation of VAD support in LVAD and BiVAD (/HeartMate II or Heartware) assisted patients [43, 44]. Taken together, EV numbers are commonly elevated in the circulation of patients who are supported by ExCor/ECLS, with transitory elevations in the case of temporary ExCor/ECLS, and subtler yet progressive increases in case of prolonged-ExCor/ECLS.

## LEVELS OF EVS ARE PROPORTIONAL TO DISEASE SEVERITY

Sick patients, such as those requiring hemodialysis, often have baseline inflammation (prior to ExCor/ECLS exposure) from chronic renal dysfunction which, independent of ExCor/ECLS use, are associated with elevated levels of circulating EVs. Even more so than in patients with CPBs, ECMO, or VADs, it is therefore critical to distinguish whether EV levels in ExCor/ECLS systems, such as with HD patients (Table 7), are elevated as a result of ExCor/ECLS adverse events or simply as a result of the underlying disease and a reflection of its severity. Of interest, healthy controls have lower levels of e-, p-, g- or PS+-EVs compared to end stage renal disease (ESRD) patients [45–53], which are in turn lower than most accounts of ESRD patients requiring HD [48, 51, 52].

As such, a current limitation of interpreting EV studies is the inability to definitively determine if EVs are related to pre-existing inflammatory conditions or the use of ExCor/ECLS systems especially when control groups (healthy individuals or patients with pre-existing inflammation without ExCor/ECLS) are not available for comparison to the ECLS/ExCor patients.

## EVS ARE ASSOCIATED WITH ADVERSE EVENTS PERTINENT TO EXCOR/ECLS USE

For biomarkers of ExCor/ECLS related adverse events, there are presently limitations to obtaining prompt meaningful estimates of ExCor/ECLS related inflammation and apoptosis or an accurate means of predicting pending complications during ExCor/ECLS use. An emerging strategy of monitoring these parameters is the study of EVs in the circulations of ExCor/ECLS coupled patients. EVs have been shown to correlate with disease severity and outcomes such as cardiovascular risk; for example, e-EVs correlate with vascular dysfunction in patients with chronic inflammation such as diabetics when compared to healthy controls [54]. E-EVs have also been shown to correlate well with mortality within a high cardiovascular risk group of end stage renal disease (ESRD) patients [55]. Beyond their association with the disease processes which lead patients to requiring ExCor/ECLS, there is growing evidence of the capacity of EVs to act as biomarkers specifically for ExCor/ECLS adverse events. E-, l-, p- and r-EVs were examined from 81 patients with ESRD dialyzed three times per week 4–6 hours per session with high permeability membranes (AN69 and polysulphone); and specifically, elevated e-EV levels emerged as a key predictor of cardiovascular mortality within this population [55]. Moreover, PS+ EV counts are higher in VAD-patients with cardiac and gastrointestinal adverse events compared with complication-free VAD patients [56]. A study of 17 patients 3 months post-VAD insertion and 10 non-VAD control patients showed that VAD patients with complications had higher PS+ EV counts compared with complication-free VAD patients which were again higher than non-VAD controls [56, 57]. Similarly, elevations of e-EVs have correlative association with adverse events such as cardio-renal complications in a variety of ExCor/ECLS [39, 47, 55, 56, 58, 59]. E-EVs have been proposed to serve as a biomarker for arterial stiffness in pediatric HD patients, as pulse wave velocity, an accepted measure of arterial stiffness, correlated with CD144+ e-EV levels that were higher in HD patients compared with non-HD controls [52]. Within HD patient populations there appears to be a prognostic relationship between elevated circulating e-EVs and endothelial damage [51, 60]. Within HD patients the early loss of vascular access due to vessel stenosis and thrombosis (adverse events of HD) correlates with further elevations beyond ESRD levels of e-EVs and p-EVs [45]. Conversely, reductions in EVs are associated with reduced adverse events, as seen in a study of 40 CPB cardiac surgery patients which showed that when EV formation was reduced by nitric oxide and iloprost there was less thrombocytopenia and reduced post-operative bleeding [61]. Together these studies demonstrate that EVs frequently correlate with adverse outcomes in ExCor/ECLS. Before EVs may be applied as biomarkers of ExCor/ECLS related adverse events, important questions need clarification to facilitate meaningful interpretation of the results such as teasing out the contributions specifically from patient related factors as opposed to ExCor/ECLS factors in generating the EV profiles seen, and the utility of specific EV subsets as biomarkers. A a challenging limitation of many ExCor/ECLS studies examining EVs is to definitively show if increases in EVs are related to simply more profound disease states within patients (leading to increased ExCor/ECLS-independent susceptibility to ExCor/ECLS-adverse events) or if increased EVs directly reflect ExCor/ECLS specific changes associated with ExCor/ECLS-adverse events? Yet, a fascinating aspect of elevated levels of EVs in patients supported by ExCor/ECLS, *potentially independent of how they are formed (patient-related vs directly ExCor/ECLS-related factors),* is their potential to agonize ExCor/ECLS-associated adverse events.

## EVS ARE MECHANISTICALLY LINKED TO ADVERSE EVENTS PERTINENT TO EXCOR/ECLS USE

**Figure 2:**
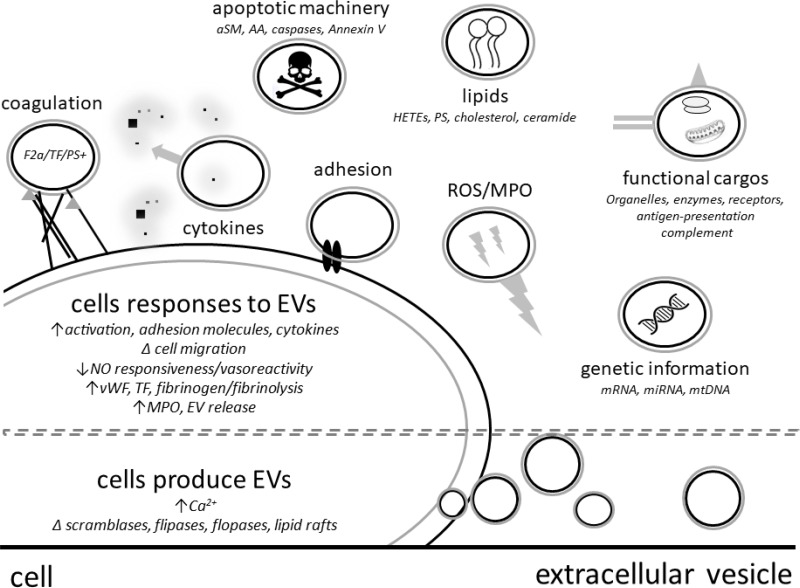
Extracellular vesicles (EVs) have direct or cell mediated effector functions such as release of cytokines, apoptosis mediators or reactive oxygen species (ROS) ABOVE DASHED LINE - EVs can contain functional cargos such as genetic material, organelles, enzymes (aSM: acid sphingomyelinase; MPO: myeloperoxidase), surface receptors (TF: tissue factor), coagulation factors (F2a: thrombin), apoptotic machinery and lipid components (PS: phosphatidylserine; AA: arachidonic acid; HETEs: Hydroxyeicosatetraenoic acids). BELOW DASHED LINE - EVs are formed from cells under conditions of stress or activation resulting changes such as increased intracellular calcium, altered activity of cell membrane symmetry enzymes such as scramblases, flippases and floppases.

Beyond considering EVs solely as potential biomarkers of ExCor/ECLS adverse events due to their association with ExCor/ECLS and associated adverse events, it is important to note that they also have the capacity to agonize processes leading to complications (Figure 2), and as such may be targets for therapeutic intervention.

EVs can influence their environment and reprogram target cells by signalling through cell receptor interactions, delivery or stimulation of release of cytokines or transferring genetic materials such as mi/mRNAs, proteins and lipids or even dock with cells or be engulfed which can change recipient cell phenotypes and functions [17, 62, 63]. By such means, EVs can influence physiologic processes during ExCor/ECLS use via a number of factors such as increasing cell-cell adhesions with endothelial cells [27, 64], activating complement (by adhering to C1q, C3 and C4) [21], increasing pro-inflammatory cytokine release (such as IL-6 [64]), transporting injurious enzymes (such as myeloperoxidase [65]), presenting antigens and adhesion factors capable of leading to *in vitro* T cell activation and proliferation [66] and in disease states reduce EV (NO) mediated vascular relaxation (Figure 2) [67–70]. As a specific example: under high shear stresses which resemble conditions in some ExCor/ECLS systems, p-EVs were shown to enhance the production of cytokines such as TNFα, IL-1β and IL-8 as well as adhesion molecules on leukocytes and endothelial cells which in turn enhances their interaction [27].

Thrombosis is a serious risk with ExCor/ECLS use and EVs can contribute in a number of ways to a pro-coagulant milieu. Endothelial damage during ExCor/ECLS use can increase expression of VWF, fibrinogen and tissue factor (TF) on circulating e-EVs [42, 71]. In addition, EVs from different cell types beyond endothelial cells can be adorned with TF and PS which additionally contribute negatively charged phospholipid surfaces and activation signals which assist in catalyzing the coagulation cascade. Along these lines, EVs derived from pericardial suctions have procoagulant activity as they were shown to enhance TF/FVII dependent thrombin formation *in vitro* [72]. Elevated levels of p- and e-EVs in HD patients have also been shown to lead to increased release of thrombin [50]. Moreover, some p-EVs have previously been shown to have potent procoagulant function described as 100-fold higher than activated platelets [73].

## STRATEGIES OF CONTROLLING LEVELS OF EVS IN EXCOR/ECLS

As evidence emerges of the ability of EVs to not only act as biomarkers but additionally mediate inflammatory processes and changes within the coagulation cascade which culminate in ExCor/ECLS adverse events strategies aiming to improve the safety of ExCor/ECLS systems should focus on minimizing the negative impact of EVs during ExCor/ECLS use. This may involve *removal of detrimental EVs, preventing EV generation, or selection for beneficial EVs.*

Specific configurations of ExCor/ECLS systems allow for *clearance of detrimental EVs. In vitro* experiments using continuous veno-veno hemodialysis (CVVH) circuits, hemofiltration circuits, apheresis machines and cell saver devices have shown capabilities to lower EVs by filtration [74–77]. Cell saver technology often used in combination with CPB in cardiac surgeries may in particular be an attractive means to efficiently remove r- and p-EVs as shown in 13 patients who underwent CPB, and in which the cell saver was able to remove more than 97% of EVs from ExCor/ECLS associated patient blood [75]. When e-EVs were placed in saline simulating a human's circulating volume and filtered with a standard CVVH 200 nm filter at flows of 250 ml/h 50% of Annexin V+ e-EVs were cleared at 30 minutes and after 4 hours there were as few as 5.7% of circulating e-EVs remaining [74]. This may be of particular interest as PS+ EVs have been shown to be associated with ExCor/ECLS adverse events [56], so selective clearance of annexin V+ EVs may be especially beneficial. E-EVs were consistently lowered post-HD in two different studies which examined 51 HD patients [53, 78]. A study examining HD patients showed indirect evidence of the ability of ExCor systems to clear EVs from the circulation by a net loss of prothrombotic activity post filtration, which returned after EVs were selectively returned to post-filtration plasmas [50]. The type of HD is an interesting area of future investigation concerning clearing EVs as there are differences in post-hemofiltration reductions in e-EVs in low-flux when compared with high-flux HD [60, 79]. HD with endogenous reinfusion appears to be a dialysis strategy capable of enhancing reduction of CD31+ Annexin V+ e-EVs from the circulation [53]. Like HD circuits, plasma-, leuko- and platelet-pheresis (apheresis) machines are capable of lowering EVs (p- and m-EVs) in the circulation of patients [76, 80, 81]. Not all apheresis studies, however, consistently show changes in levels of EVs (Table 8), as other studies have shown specifically no significant differences between p-EV levels before and after filtration [82]. The authors theorized that though the apheresis circuit may have filtered many p-EVs more may have been created due to platelet activation by the circuit. Notably, however, these EV levels were measured by ELISA and not flow cytometry which offers greater sensitivity to detect differences in numbers of p-EVs. Moreover, different configurations may impact platelet activation or the ability to filter EVs, as it has been shown that different apheresis ExCor systems or use of single compared with double needle systems within a single ExCor system influence EV counts [83]. Similarly, the type of materials – including differences in pore volumes, surface area and charge density - used in absorbers in apheresis machines influences which cells adhere and EV levels [84]. Cell-adhesiveness of filters/absorbent polymers may influence which types of cells adhere, which in turn may influence EV generation from these cells. Small pore sizes such as 75 nm, can lead to removal of EVs, while preserving proteins, lipoproteins and coagulation factors [85]. Over and above that, not all ExCor systems may be able to efficiently filter uremic toxins, which may impact EV levels. Some uremic toxins such as indoxyl sulphate and p-cresyl sulphate are not universally cleared by ExCor systems such as HD and can contribute to e-EV production [46, 68]. As such Faure and coworkers noted that un-dialyzed chronic renal failure (CRF) patients had similar levels of e-EVs as dialysis patients possibly due to similar uremic burdens between these groups [46]. This concept is corroborated by the finding that *in vitro* stimulation of human umbilical vascular endothelial cells with uremic toxins elicits e-EV formation [46]. In rheumatoid arthritis patients leukopheresis removed p-EVs and increased anti-inflammatory g-EVs, suggesting that different EV subsets may be individually modulated [80]. These studies provide important insights of how select ExCor/ECLS systems with specific configurations may deplete circulating EVs and may guide the way to the design of modified ExCor/ECLS systems optimized for EV removal. To this end, it is particularly advantageous that selectivity appears obtainable, as seen by filters (75 nm) capable of reducing prothrombogenic EVs but preserving coagulation function in plasma samples [85]; as sequestration and removal of too many of the constituents of the coagulation cascade could lead to significant coagulopathy. Further investigations are urgently needed to test the consequences and thus potential applications of EV removal in patients by filtration with ExCor/ECLS systems.

Pharmacologic agents may be able to *reduce EV formation* which in turn may be beneficial in reducing adverse events associated with ExCor/ECLS use. ExCor/ECLS systems enriched with nitric oxide (20 ppm) or prostaglandins (iloprost (2 ng/kg/min)) reduce platelet activation and prevent p-EV formation [61]. While these effects were associated with less post-operative chest tube bleeding, a direct causative link between the reduced p-EV formation and the improved coagulation state remains to be shown [61]. Other cardiovascular medications may also have beneficial effects on circulating EV levels as there is growing evidence that angiotensin-receptor blockers and HMG-CoA reductase inhibitors (statins) can lower p-EVs in ESRD patients [50] and m-EVs in hypertensive or diabetic patients [86]. Similarly, statins have been shown to reduce e-EV formation from TNFα stimulated human coronary endothelial cells *in vitro* [87]. During CPB, elevations of p-EVs can be prevented by treating platelets with GP2α3β inhibitors and heparin [88], and at the experimental level, agents such as caspase-, PI3K- and P(2)Y-inhibitors further reduce platelet activation and p-EV formation [89, 90]. Considering these medications for the goal of reducing EVs would, however, require careful attention to the specificity of their effects and potential adverse events such as bleeding or platelet imbibition. A future area of investigation may include a means of mitigating the systemic effects of these medications with strategies such as having them coated on ExCor/ECLS surfaces instead of systemic administration into the patient's circulation. Preliminary tissue engineering experiments involving lining oxygenator membranes under static conditions with fibronectin have shown preferential adherence of circulating endothelial cells [91]. Lining circuits with endothelial cells may help reduce foreign material exposure to blood and lower EV formation due to decreased cellular activation and inflammation in patients requiring long term ExCor/ECLS support such as those having destination therapy VADs. At the same time, however, an endothelial cell lining on ExCor/ECLS systems may become a relevant source of e-EV production due to high shear forces generated by blood circulating within ExCor/ECLS systems. Beyond lining circuits with fibronectin or heparin, it may be possible to line sections of extracorporeal circuits with immobilized antibodies (such as IgM [92]) or other adhesive molecules to selectively sequester and eliminate EVs within patients connected to ExCor/ECLS systems. Beyond parenteral or enteral route medications, oxygen tension may impact EV numbers in patients with ExCor/ECLS systems. Hyperoxia has the potential for EV formation in part due to neutrophil and platelet activation, aggregation and reactive oxygen species (ROS) production [93, 94] and vice versa hypoxia is a known trigger for EV formation [95]. Strict regulation of partial pressures of oxygen may thus prove an accessible strategy to reduce EVs. Taken together, existing and experimental drugs, bioengineering approaches or revised SOPs for ExCor/ECLS patient care such as adhesion to normoxic ventilation may offer potential means of reducing circulating EVs passing through ExCor/ECLS systems.

Lastly, not all EVs are detrimental, and as such should not necessarily be removed from the circulation. Of the EV populations that may have *therapeutic potential,* endothelial progenitor cell (EPC)-EVs and mesenchymal stem cell (MSC)-EVs show presently the most promise. EPC-EVs improve ischemic renal injury [96] which is a complication relevant to ExCor/ECLS use. Erythropoietin treatment in ESRD patients dose dependently boosts MSC-EV formation which proved beneficial in animal models of renal injury [97]. Beneficial effects may, however, not be limited to stem- or progenitor-cell derived EVs. Though e-EVs from diseased patients can do harm as outlined in previous sections of this review, e-EVs from healthy individuals can transport eNOS which may be able to boost intravascular NO generation [70]. Under normal/healthy conditions e-EVs carry functional eNOS, whereas during states of disease, e-EV eNOS expression is lost which leads to less NO formation which is a pro-relaxant mediator for vascular/endothelial tone. As such selective delivery of healthy e-EVs may be beneficial to certain patients with ExCor/ECLS systems. In order for EVs to become a cell-free therapeutic option, more studies are needed to decipher which EVs may broker benefits as opposed to harms, and strategies are required to concentrate beneficial EVs while removing harmful EVs.

## TECHNICAL CHALLENGES OF USING EVS AS BIOMARKERS OF EXCOR/ECLS ADVERSE EVENTS

As the study of EVs as biomarkers and effectors of disease evolves, hurdles remain such as limitations in strategies for their isolation and accurate characterization. Challenges associated with reproducible collection and analysis of EVs are summarized in a recent review [98]. EVs exist in many biological fluids. The most popular site for monitoring EVs pertinent to ExCor/ECLS use is blood. In order to optimally measure EVs in anticoagulated blood samples, it is recommended to rapidly (<1 hour) remove cells and debris by differential centrifugation, which may lead to confounding false positive counting of non-EVs such as seen with small platelets, or even lead to post-collection generation of EVs if cells activate during *in vitro* processing [99]. In most ExCor/ECLS studies, EVs are measured by flow cytometry which offers advantages of being accessible and allowing high throughput analysis of many EVs. Alternative high throughput EV analysis tools exist such as Nanotracker technology, mass spectroscopy and tunable resistive pulse sensing. Other complimentary techniques for EV characterization comprise functional assays to assess EV surface receptors or procoagulant capacity using enzyme linked immunosorbent assays or single EV analyses with electron and confocal laser microscopy. Interpretation of EV studies can be complicated, though, as analytical tools for EV detection such as flow cytometers (the most popular detection device for ExCor/ECLS EV studies) function at their lower limit of detection to measure small EVs which can be confounded by instrument noise or debris, while at the upper size limit measurements can be complicated by the presence of platelets or EV-platelet aggregates. Beyond aggregation which can lead to inaccurate detection of EVs, fusion or adherence of MPs to other EVs or cells has been reported in ExCor/ECLS EV studies which add further challenges to enumerating EVs [3, 37, 48, 100]. Taken together, while use of EVs as biomarkers is rapidly evolving, the basic methodology for their standardized assessment is still under development. Conventions and guidelines for acquisition, handling and interpretation of EVs from ExCor/ECLS studies are needed.

## UNKNOWNS AND FUTURE DIRECTIONS

Beyond the ExCor/ECLS configurations discussed in this review, there are others that have not yet been assessed in terms of their effects on EV formation and characteristics, such as small circulatory assist devices like the impala or *ex vivo* solid organ transplant perfusion circuits. An emerging example of novel ExCor/ECLS systems are experimental or clinical *ex vivo* organ perfusion systems for lung, heart, kidney and liver [101–104] which remain largely unstudied. Beyond different types of circuits, this review did not address the possible presence and contribution of exosomes and apoptotic bodies, which may be important in terms of biomarkers or pathomechanistic effectors. Yet there is presently a paucity of publications examining these EVs in the context of ExCor/ECLS use which thus presents an important area for future investigation. An exciting aspect of research is assessing the impact of EVs seen in ExCor/ECLS circuits in terms of their capacity to mediate disease processes and accordingly, the implementation of EV targeted strategies in ExCor/ECLS systems to alleviate these detrimental adverse effects.

## CONCLUSIONS

Most ExCor/ECLS systems (acute- or prolonged-support) lead to transient or permanently increased levels of circulating EVs. This had fueled the idea that they may be exploited as biomarkers for prognosis or indicators of potential ExCor/ECLS-related adverse effects; but recently also generated interest in the role of EVs as propagators of disease processes and mechanisms. Within ExCor/ECLS circuits certain components such as oxygenators regionally agonize EV formation. Levels of circulating EVs increase primarily during ExCor/ECLS use, and return to lower levels with discontinuation of circuit use. When characterizing the contribution of ExCor/ECLS systems to elevated EV levels from patients, it is, however, important to consider the basal levels of EVs which may be elevated prior to ExCor/ECLS exposure due to underlying diseases and comorbidities. EVs offer potential as biomarkers to predict adverse events associated with ExCor/ECLS use, and monitoring abundance, time profiles, and antigenicity of EVs has potential to become implemented as routine screening and monitoring tool into clinical practice. The most numerous EVs in circulation appear to be p-EVs. The presence of PS+, p- or e-EVs each can correlate to adverse events related to use of different ExCor/ECLS systems.

New insights and opportunities are emerging which may improve care for patients requiring ExCor/ECLS use. Though many ExCor/ECLS systems appear to agonize EV production it seems select ExCor/ECLS sytems may offer capacity to reduce their levels. Manipulation of EV numbers or subtypes may be of use for reducing disease and improving coagulation status in the context of anti-coagulated patients paired with ExCor/ECLS. Future ExCor/ECLS designs may involve optimization for controlling EV levels and subtypes. These opportunities are not without risk. EVs, as described above can have both beneficial and detrimental effects in biology. The consequences of indiscriminate or selective reduction/removal of EVs or their manipulation is not well understood at present, in terms of physiological changes.

Manipulation of EVs may be of particular interest in oncology, where they are associated with dissemination of metastatic disease [19]. Cancer cell EVs can lead to immunomodulation and promote tumor growth and metastasis, therefore developing strategies such as ExCor/ECLS systems to remove unwanted EVs could be an attractive adjunct strategy when treating cancer patients [19, 77]. Though typically, advanced metastatic disease is a contraindication to ExCor/ECLS use, employing ExCor/ECLS to reduce EVs may allow reduced progression of metastases and prolong quality of life. Boosting beneficial EV subpopulations such as EPC- or MSC-EVs may be an even more ambitious, but ultimately rewarding strategy with the aim to establish beneficial EVs as cell-free cell therapeutics the use of which would not be limited to ExCor/ECLS.
